# Case report: Delayed quadriplegia from traumatic carotid cavernous fistula: a rare case with perimedullary venous drainage

**DOI:** 10.3389/fneur.2023.1224425

**Published:** 2023-08-21

**Authors:** Yu-Hu Ma, Rui Shang, Sen Lin, Si-Hao Li, Ting Wang, Chang-Wei Zhang

**Affiliations:** Department of Neurosurgery, West China Hospital, Sichuan University, Chengdu, Sichuan, China

**Keywords:** carotid cavernous fistula, perimedullary drainage, quadriplegia, coil embolization, case report

## Abstract

**Background:**

Carotid cavernous fistula (CCF) refers to the abnormal arteriovenous communication between the carotid system at the skull base and the sphenoid cavernous sinus, which is caused by trauma in almost 75% of cases. The drainage of venous blood to the spinal cord represents a distinctive mechanism, which is commonly observed in dural arteriovenous fistula (DAVF), and typically manifests clinically as progressive myelopathy. However, it is a rare occurrence in clinical practice that traumatic carotid cavernous fistula (TCCF) causes delayed quadriplegia through perimedullary venous drainage.

**Case presentation:**

We report the case of a 29-year-old male patient who was admitted to the hospital with a sudden onset of headache and quadriplegia. The patient had previously lost his right eye in a traffic accident 5 years ago. Cerebral angiography showed a high-flow direct CCF on the right side, accompanied by obvious drainage of cerebellar and perimedullary veins. We successfully performed coil embolization for the CCF, and the symptoms of the patient gradually improved after the operation. During follow-up at sixth-months, the patient regained the ability to walk independently.

**Conclusion:**

We experienced a rare case of TCCF with quadriplegia. Utilizing coil embolization, we achieved successful improvement in the patient’s condition. However, the mechanism and the best treatment of CCF drainage through the perimedullary vein are still unclear. We need to further explore the pathophysiological information of CCF venous drainage.

## Introduction

Carotid cavernous fistula (CCF) refers to the abnormal arteriovenous communication between the carotid system at the skull base and the sphenoid cavernous sinus, usually manifested as pulsatile exophthalmos, conjunctival congestion, and intracranial murmur (1). CCF is a rare but not unique disease, with traumatic causes being the most common, accounting for approximately 75% of cases (2). Traumatic carotid cavernous fistula (TCCF) only occurs in 0.2% of the brain or in maxillofacial trauma (3) clinically, and the symptoms largely depend on the direction of venous drainage of the cavernous sinus (4, 5). While venous blood flowing to the spinal cord is a rare drainage method commonly observed in dural arteriovenous fistula (DAVF), its clinical manifestations are predominantly progressive myelopathy. According to the classification scheme proposed by Cognard et al., this type of vascular disease is classified as Cognard V (6). However, there are few cases of tetraplegia caused by TCCF, resulting in limited information regarding its mechanism. This manuscript describes a case of TCCF presenting as quadriplegia through intraspinal drainage.

### Case presentation

A 29-year-old male patient was admitted due to an acute onset of severe headache and quadriplegia. The patient’s medical history can be traced back to a traffic accident five years ago, during which he sustained head and abdominal injuries. Although a cranial computed tomography (CT) scan performed at the time of the accident showed no abnormality, the accident eventually led to blindness in the patient’s right eye. Over the past five years, the patient has remained asymptomatic with no clinical manifestations.

**Figure 1:**
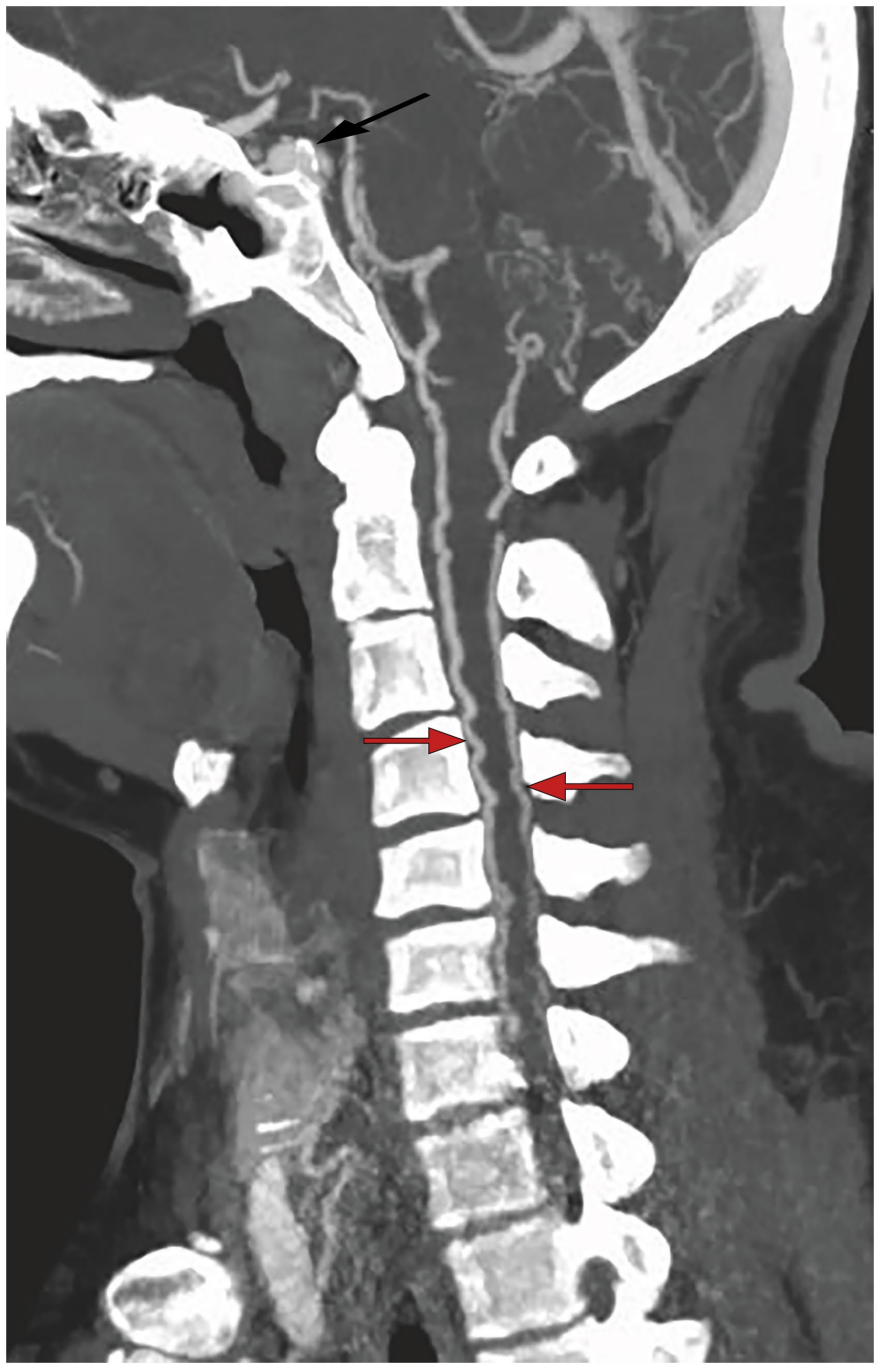
Preoperative CTA imaging showed that there was an abnormal vascular mass in the right cavernous sinus area, and prominent vascular structures could be seen around the medulla.

**Figure 2:**
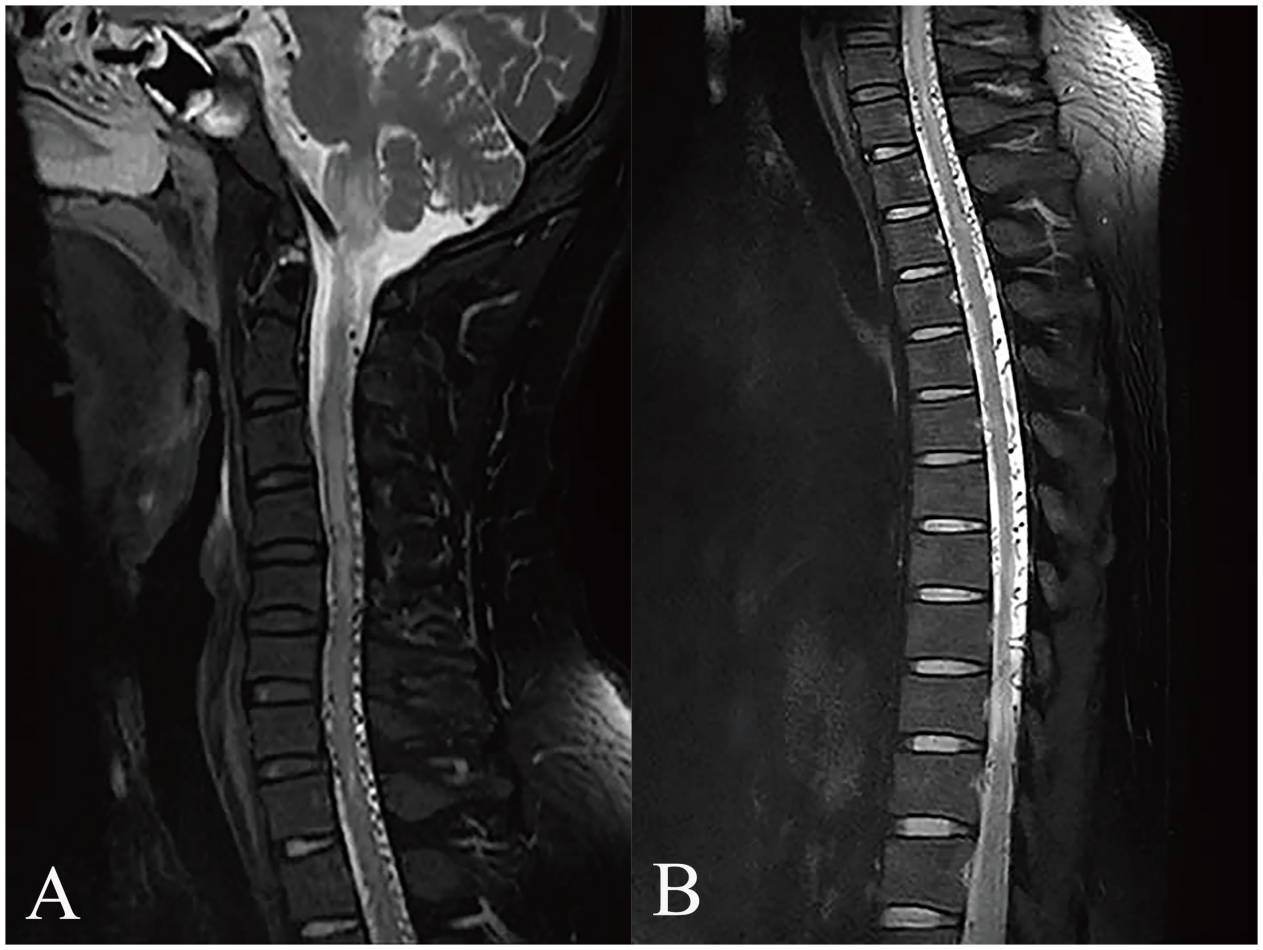
MRI examination of the affected region. The MRI scan reveals medullary and cervical cord edema with multiple tortuous vascular flow voids **(A)**. Additionally, tortuous vascular flow voids are also observed in the thoracic and lumbar cord regions **(B)**.

**Figure 3:**
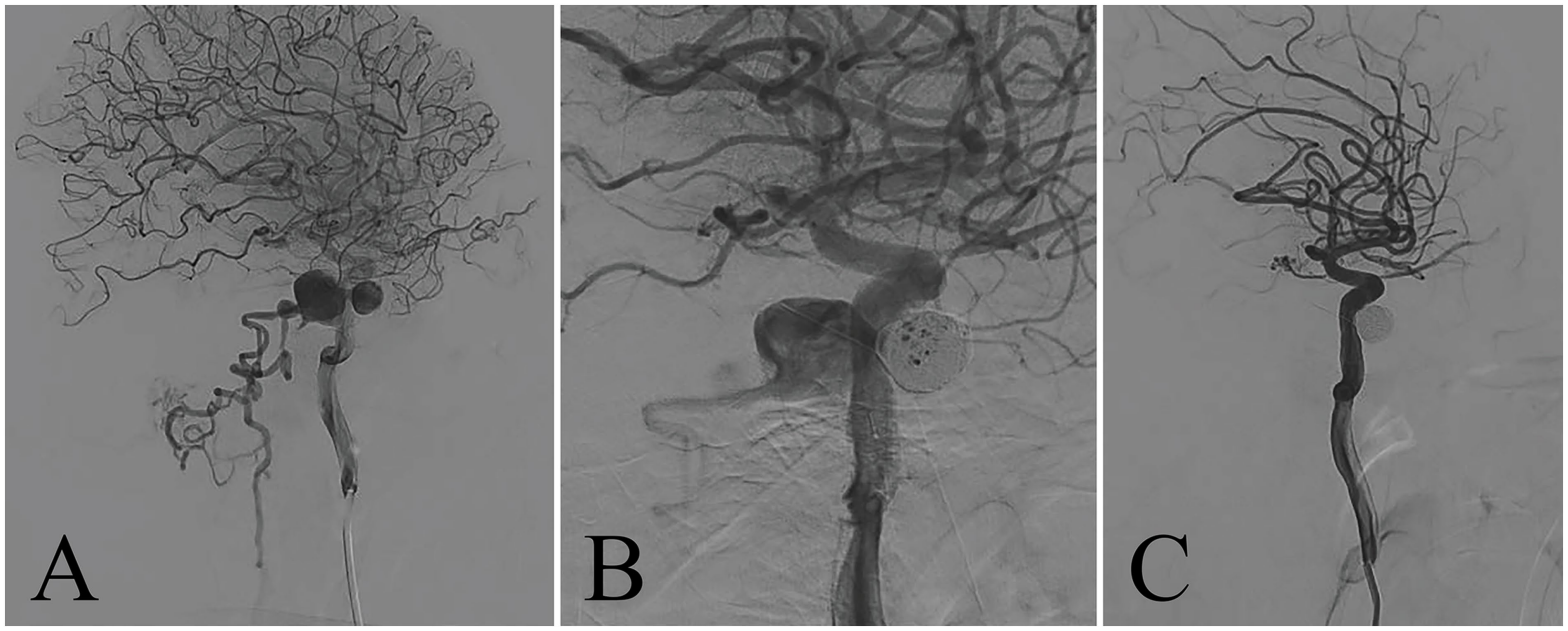
Angiography of right internal carotid artery. The preoperative DSA showed that the cavernous sinus was abnormally filled and blood flowed back through the perimedullary vein **(A)**. Intraoperative DSA for coil embolization **(B)**. After embolization of the cavernous sinus with arterial coil, the fistula was completely occluded **(C)**.

The patient was admitted to West China Hospital of Sichuan University (Chengdu, China) through the emergency department, presenting with an abrupt onset of severe headache and bilateral lower limb paralysis upon awakening during the night. A comprehensive neurological assessment of the patient elucidates right-sided blindness, graded bilateral upper limb muscle strength of 2, and graded bilateral lower limb muscle strength of 0. The patient demonstrated reduced upper limb tendon reflexes and a complete absence of knee and ankle reflexes in both lower limbs, concurrently presenting positive pathological indicators bilaterally. Upon admission, an urgent cranial CT scan showed no significant abnormalities, while head and neck computed tomography angiography (CTA) demonstrated an anomalous vascular mass in the right cavernous sinus area, contiguous with the internal carotid artery (ICA), accompanied by dilated and tortuous perimedullary vascular structures (Figure 1). The patient’s spinal cord magnetic resonance imaging (MRI) examination revealed medullary and cervical cord edema, along with multiple tortuous vascular flow voids (Figure 2). These findings raised the suspicion of a carotid cavernous fistula (CCF). Subsequently, a cerebral angiography was expeditiously conducted, with the digital subtraction angiography (DSA) revealing a high-flow CCF on the right side draining into the intraspinal venous plexus and cerebellar vein (Figure 3A). The CCF’s drainage into the spinal canal led to spinal cord edema and ischemia, providing an explanation for the patient’s limb muscle weakness symptoms. Then contrast agent was injected into the external carotid artery, and no dural vessel branches supplying the CCF were found (consistent with Barrow Type A CCF). The patient was diagnosed with a traumatic carotid cavernous fistula (TCCF).

Based on the analysis of cerebral angiography, the treatment plan for this case was scheduled for right CCF coil embolization *via* femoral artery access. Prior to the procedure, a traditional femoral artery puncture was performed, and systemic heparinization was administered to prevent thrombus formation during the intervention. Subsequently, a neurovascular guidewire was used to guide the placement of the catheter into the petrous segment of the ICA, ensuring accurate positioning for subsequent steps. During the surgery, intraoperative DSA was employed to confirm the precise location of the fistula’s opening. With the assistance of the micro guidewire, the microcatheter was carefully advanced to the site of the CCF’s orifice. Utilizing precise placement, five coils were deployed to embolize and effectively occlude the abnormal arteriovenous communication (Figure 3B). The entire procedure was smoothly executed, and immediate post-interventional DSA demonstrated successful closure of the fistula, absence of draining veins, and preservation of the integrity of the ICA (Figure 3C).

The patient’s postoperative recovery displayed promising improvements. On the third day after the operation, the patient experienced a noticeable reduction in headache intensity, along with improvement in upper limb muscle strength, and slight toe movement. At the 1 month follow-up, the patient’s limb strength continued to improve gradually, with the ability to walk assisted by crutches. Encouragingly, during the 6-month follow-up after the operation, the patient’s upper limb muscle strength had essentially returned to normal, and both legs were capable of slow independent walking, significantly enhancing the patient’s quality of life.

## Discussion

Traumatic carotid cavernous fistula (TCCF) is a rare complication of craniofacial trauma. It was first reported in 1835, and is mostly seen in middle-aged males. Incidents of forceful trauma can result in fractures of the skull base. The fracture fragment may directly perforate, displace, or tear the internal carotid cavernous sinus (CS) segment, leading to the rapid ingress of arterial blood into the cavernous sinus and the consequent formation of TCCF (7). In 1985, Barrow et al. (8) classified CCF into four types (type A to type D) based on the arterial architecture. TCCF represents a direct communication between the C4 segment of the internal carotid artery (ICA) and the CS, which usually belongs to high flow disease. When TCCF occurs, arterial blood tends to flow rapidly into the cavernous sinus, which increases the pressure in the sinus. Consequently, The hemodynamic alterations in the CS promote retrograde or clockwise blood flow through multiple veins (9). In 1972, Houser et al. (10) initially proposed that the clinical manifestations of CCF mainly depend on the direction of blood drainage within the CS. Diverse drainage patterns result in distinct clinical symptoms.

Typically, venous drainage patterns of TCCF can be categorized into the following four groups (11):

(1) Anterior Drainage: It involves forward flow toward the ophthalmic vein, medial canthus vein, or facial vein, eventually reaching the external jugular vein. Most CCF cases are drained to the ophthalmic vein (the most typical venous drainage pattern), resulting in the ophthalmic triad (consisting of exophthalmos, bulbar conjunctival edema, and orbital murmur) corresponding to CCF (12). (2) Posterior Drainage: The fistula may drain backward toward the superior petrosal sinus or inferior petrosal sinus, finally emptying into the internal jugular vein and draining to the pterygoid plexus through a guide vein (13). Imaging studies have demonstrated a significant increase in the flow velocity of the internal jugular vein in such cases. (3) Upward Drainage: It is drained upward to the cerebral cortex or deep vein through the lateral fissure vein. The clinical manifestations often include headaches, increased intracranial pressure, and even subarachnoid hemorrhage. (4) Contralateral Drainage: In certain instances, drainage occurs through the contralateral CS, leading to bilateral pulsating exophthalmos (14). However, it is extremely rare for TCCF to result in quadriplegia through intraspinal drainage, and only 3 cases have been reported so far (15–17). Given the unclear pathogenesis and prognosis of this particular case, we conducted a comprehensive review of the clinical characteristics of this case and the three cases previously reported in the literature.

Ricolfi. F et al. (15) described the first case of CCF with perimedullary drainage leading to myelopathy in 1999. In this case, the patient had tetrapareis, sphincter disturbance, and bulbar signs at admission. MRI revealed perimedullary vessels, and high signal intensity in the swollen medulla and cervical cord. Selective angiography demonstrated a right-sided CCF supplied by meningeal branches of the ICA and external carotid artery (ECA), draining into the superior ophthalmic vein anteriorly and posteriorly into the cervical spinal cord veins via the superior petrosal sinus and lateral mesencephalic veins. In this case, they chose to take an embolization treatment by mixing a mixture of histoacryl and lipiodol in the middle meningeal and sphenopalatine arteries and polyvinyl alcohol foam particles (PVA) particles in the ascending pharyngeal artery. However, no anticoagulant therapy was administered after embolization. This patient’s neurological condition rapidly deteriorated and they passed away 5 days later. A study published in 2011 described another case of TCCF with perimedullary drainage (16). This patient had a clear history of trauma and presented to the hospital with tetraparesis. Angiography revealed a direct CCF with prominent pontine mesencephalic and perimedullary venous drainage. The physician performed coil embolization of the TCCF, which successfully improved the patient’s symptoms to walk with the aid of external assistance. Two years ago, a study (17) reported the third case of TCCF with perimedullary venous drainage. In this case, the patient developed progressive gait disturbance, hyperreflexia, hypoesthesia, and pulsatile tinnitus one month after an anterior skull base fracture. Cervical MRI revealed spinal cord edema and serpentine signal flow voids, which were eventually confirmed to be TCCF with drainage into the perimedullary veins through DSA. The treatment approach chosen by the physician involved the combined use of coils and the Onyx liquid embolic system to occlude the fistula. Postoperatively, the patient experienced the complete resolution of symptoms.

In our presented case, the patient had significant spinal cord symptoms with progressive aggravation. Cerebral angiography confirmed a high-flow CCF on the right side with drainage into the spinal canal and the cerebellar veins. It is noteworthy that the patient’s right eye was blind from trauma 5 years ago and the left eye did not show any abnormal symptoms and signs. We finally opted to perform TCCF embolization using coils. Postoperatively, at the six-month follow-up, the patient demonstrated remarkable progress and was able to walk independently.

Venous drainage to the spinal cord is a recognized drainage modality for DAVF, first proposed by Woimant et al. (18) in 1982. Since its initial description, several case reports and case series have provided detailed accounts of this vascular condition (19–21). According to the classification by Cognard et al. (6), this particular vasculopathy has been classified as type V based on clinical and angiographic correlation. Patients with Cognard type V DAVF often present with non-specific clinical symptoms. Due to elevated spinal venous pressure, 62% of patients exhibit progressive myelopathy, while 31% display bulbar dysfunction associated with dysautonomic signs (22). In addition, it is difficult to make a timely diagnosis of Cognard type V DAVF in clinical practice because the MRI findings of lesions in this category may resemble those seen in inflammatory demyelinating diseases, infarctions, and intramedullary tumors. Here, we report a case of TCCF presenting as tetraplegia through intramedullary drainage. It is of concern that the two reported cases in this context had eye-related symptoms prior to the onset of spinal cord symptoms. In the first case, treatment initially involved embolization because of moderate right-sided proptosis with conjunctival hyperemia, associated with a spontaneous CCF. The identification of CCF with perimedullary drainage occurred only 1 year later. The second patient was extremely similar to our case, also suffering from blindness in the right eye due to a history of trauma. This suggests that the occurrence of the rare perimedullary drainage pattern in CCF may be related to abnormal ophthalmic vein function, leading to a failure in proper drainage through this vein.

We hypothesize that the patient’s traumatic accident causing blindness in the right eye 5 years ago may have resulted in impaired ocular venous circulation, gradually leading to spontaneous thrombosis. Following the discovery of the TCCF, the arterial blood flow directly bypasses the fistula ostium into the CS. The massive shunting of blood flow from the ICA leads to the reduction of blood supply to the affected intracranial artery and the opening of collateral circulation.

The venous system, on the other hand, has increased blood flow and elevated pressure, leading to the development of abnormal drainage channels. Venous blood within the CS begins to drain toward the cerebellar veins and intraspinal region due to obstruction caused by spontaneous thrombosis in the ophthalmic veins. Thus, abnormal perimedullary veins were clearly visualized on CTA imaging in this patient. Escalated blood flow and increased venous pressure in the perimedullary veins gradually led to venous dilation, causing spinal venous hypertension syndrome. This explains the patient’s initial presentation of progressive quadriplegia. A study by Hassler et al. (23) demonstrated that the pressure on the intradural perimedullary vein was 60–87% higher than the mean systemic arterial blood pressure in spinal DAVF surgery. Because of the lack of valves, the intramedullary veins are directly affected by the increased pressure in the perimedullary venous plexus. Consequently, arteriovenous pressure gradients within the spinal cord decrease, leading to the development of extracellular edema and congestive myelopathy. This provided a theoretical basis for our conjecture.

Undoubtedly, this case has introduced a novel perspective for clinicians, emphasizing the importance of considering the possibility of progressive myelopathy arising from intracranial vascular malformations. Currently, due to the rarity of this disease, a significant proportion of clinicians lack familiarity and precious therapeutic experience in managing it. However, it is crucial not to overlook the potentially life-threatening nature of TCCF through perimedullary venous drainage, underscoring the significance of early recognition, diagnosis, and treatment. We firmly advocate that CCF with venous drainage into the brainstem, cerebellum, and spinal cord should be included in the differential diagnosis of progressive myelopathy, contributing to early diagnosis and optimizing patient outcomes. In addition, in future instances resembling these cases, particular attention should be paid to the patient’s ocular symptoms and the reflux of ocular veins, as such observations might offer valuable insights into the underlying mechanism of these cases.

## Conclusion

We encountered an exceptional and rare case of TCCF with quadriplegia. The application of coil embolization demonstrated notable efficacy in improving the patient’s condition. However, the precise mechanism and optimal treatment strategy for CCF drainage through the perimedullary vein remain enigmatic. Further exploration of the pathophysiological aspects related to CCF venous drainage is imperative to gain a comprehensive understanding and valuable insights.

## Data availability statement

The original contributions presented in the study are included in the article/supplementary material, further inquiries can be directed to the corresponding author.

## Ethics statement

Written informed consent was obtained from the individual(s) for the publication of any potentially identifiable images or data included in this article.

## Author contributions

Y-HM wrote the manuscript and edited the images of the article. Y-HM, RS, and S-HL revised the existing literature together. Y-HM, SL, and TW collected patient data and provide corresponding explanations. C-WZ completed the surgery and designed this study. All authors have contributed to the article and approved the submitted version.

## Conflict of interest

The authors declare that the research was conducted in the absence of any commercial or financial relationships that could be construed as a potential conflict of interest.

## Publisher’s note

All claims expressed in this article are solely those of the authors and do not necessarily represent those of their affiliated organizations, or those of the publisher, the editors and the reviewers. Any product that may be evaluated in this article, or claim that may be made by its manufacturer, is not guaranteed or endorsed by the publisher.
